# Supplementary material for: Detecting Long-Term Balancing Selection Using Allele Frequency Correlation
Source: Mol Biol Evol. 2017 Jul 21;34(11):2996–3005. doi: 10.1093/molbev/msx209 (PMC5850717; doi:10.1093/molbev/msx209)
Supplement: Supplementary Data [file msx209_supp.pdf]

· doi:10.1093/molbev/sys000 MBE

METHODS

Power Analysis

To calculate the power of each method, we compared the score of the balanced variant in balanced simulations with the score of SNPs matched for equilibrium frequency in neutral simulations. For each neutral simulation replicate, we randomly identified one SNP in the simulated region at a frequency within 10 percent of the equilibrium frequency of the corresponding simulations with a balanced SNP. Throughout our discussion of simulations, we refer to the number of the haploid genotypes, corresponding to the total number of chromosomes, as the number of individuals. Power calculations were performed with p=20 for Beta, and w=10 for T1 and T2, unless otherwise specified.

The T1 and T2 statistics require an estimate of divergence time with the outgroup species and a summary of the background levels of polymorphisms and substitutions. To generate these empirical genome-wide estimates, we pooled all of our neutral simulation replicates for the appropriate parameter set, and then fed these into the functions provided by BALLET, the software package implementing the T1 and T2 statistics (DeGiorgio *et al.*, 2014).

To generate expectation and variance for the HKA test, we took 1kb regions from each of our neutral simulations under the relevant parameter set. We then calculated the mean and variance of the number of sites that are polymorphic in the human simulated population, and of the average number of differences between a random human individual and the chimp outgroup individual. Our HKA statistic was then the sum of two chi-squared statistics: one corresponding to the number of human polymorphisms, and one corresponding to the average number of human/chimp differences.

For the mutation and recombination rate variation power analysis, we used the background files generated using the simulations based on our default rates. The reason for this is to both check for over-fitting to these parameters and also to test for power upon misspecification of population parameters.

CHOICE OF P PARAMETER

The power of our method lies in capturing allele frequency correlations. The parameter p controls how similar of allele frequencies to the core site are captured. As p approaches infinity, the only sites that contribute towards θ_B are those that exactly match the frequency of the core SNP. At $p=0$, all SNPs contribute the same amount to the estimate of $\hat{\theta}_B$, and so $\hat{\theta}_B$ becomes equivalent to $\hat{\theta}_w$. Simulations show that our method is fairly robust to choice of p (**Fig. S12**).

That said, the optimal p will depend on the data set at hand. If allele frequency estimates are known to be inaccurate or sample sizes vary between SNPs, then a lower p may be more optimal, because variants fixed in allelic class may

not accurately be called as being at identical frequency to the core SNP. In addition, allowing variants at very similar frequency to the core SNP contribute to $\hat{\theta}_B$ allows it to capture SNPs that are very close to fixing in allelic class, or were once fixed in class, but are no longer due to recombination followed by a small amount of drift. However, making p too low will result in the inclusion of allele frequencies that are very different than the balanced allele's frequency. In our analysis, we chose a $p = 20$, which gives the most weight to exact frequency matches, and a small amount of weight to very near, but not exact frequencies. If varying sample sizes are used for each SNP, then a lower p value is optimal (**Fig. S11**).

SUBSTITUTIONS AND β

We also wanted to explore whether taking the number of fixed differences with an outgroup (substitutions) would increase power. Substitutions are used by the HKA, T1 and T2 tests. However, we observed that the number of substitutions does not greatly increase predictive capabilities over that of just β values (AIC with logistic regression of 2523 with just Beta values, 6246 with just substitutions and 2434 combined). Thus, we decided to focus our method only on polymorphism data.

SIZE OF THE ANCESTRAL REGION

In order to generate a theoretical expectation for the optimal window size, we want to derive the expected length of the ancestral region around the balanced SNP, i.e., what is the distribution of region sizes around the balanced site where the coalescent tree looks identical to that of the balanced loci, ignoring mutation. The ancestral region is the region starting at the balanced variant, moving outwards in either direction until an observable recombination event has occurred in the history of the sample. An observable recombination event is one in which there was recombination between allelic classes. This concept is similar to that in Gao *et al.* (2015); however, we are not concerned with an outgroup species, which simplifies the derivation.

The ancestral region roughly corresponds to the optimal window size to calculate β on, because it contains the region in which alleles can fix in allelic class, and have not been decoupled from selection due to recombination. In reality, this may slightly underestimate the optimal window size, because it is possible for a position to "re-fix" in allelic class. In this scenario, a recombination event occurred, then a new mutation arose and drifted up to the balanced frequency.

The probability of recombination between allelic classes is equal to the total coalescent branch length in the allelic class multiplied by the probability of recombination onto the other allelic class. Because we are detecting long-term selection, most of the coalescent branch length will fall into the portion between coalescence within each allelic class and coalescence of the two allelic

· doi:10.1093/molbev/sys000 MBE

classes. We can, therefore, put an upper bound on the size of the ancestral region. The probability of any recombination event occurring at a certain position at any time point in T generations is ρ*T, where ρ is the individual recombination rate. The probability of a recombination event occurring between a chromosome from allelic class 1 and any chromosome from allelic class 2, given that a recombination event occurs in a chromosome from class 1, is just the frequency of allelic class 2. Similarly, the probability that if a recombination event occurs in class 2, it is with any chromosome from class 1 is just the frequency of allelic class 1. Let λ be the rate of observable recombination, in units of base pairs, where p and q are the frequencies of the 2 allelic classes, which must sum to 1 by definition.

$$\lambda = T\rho p + T\rho q$$

$$\lambda = T\rho$$

The distribution of ancestral segments on either side of the balanced loci is then exponential with rate parameter $T\rho$.

For our analysis of the 1000 Genomes Project, we are focusing on detecting events that occurred after a split with chimpanzee, but that are old enough that our method has power. Assuming a recombination rate of 2.5e-8 and a split time of 250,000 generations prior with selection starting at this same time, the 95th quantile on either side is then 479. The most recent events we can hope to detect are closer to 100,000 generations prior to present, giving a 95th quantile of 1198 bases on either side of the core SNP. Based on these estimates, we chose to perform our analysis using a window size of 500 base pairs on either side of the core site, for a total size of 1kb.

MATCHING THE NUMBER OF INFORMATIVE SITES TO WINDOW SIZE

The T1 and T2 statistics determine window size in units of the number of informative sites (polymorphisms plus substitutions) on either side of the core site. In contrast, the other methods (HKA, Beta and Tajima's D) require a sliding window length in number of base pairs. Therefore, in order to compare the methods as fairly as possible, we derive the number of informative sites expected in the 1 kb window we use for the HKA, Beta and Tajima's D statistics.

The number of informative sites is equal to the number of substitutions plus the number of polymorphic sites. Let E[K] be the expected number of substitutions, and let E[S] be the expected number of segregating sites. We assume no recombination at this locus, because 1kb is roughly the size of the ancestral region (see previous section: Size of the Ancestral Region). Let T be the TMRCA of the two species (250,000 generations in our case), and let U be the time at which balancing selection started. Let $L_{n>1}$ be the length of the coalescent tree of a single allelic class when there is more than one lineage still in

existence in that class, and let $H_{n>1}$ be the height of the tree where this same condition holds true. Let $L_{n\geq 1}$ be the total length of the coalescent tree for a given allelic class, which begins at the start of selection. For simplicity, we assume $T \geq U$, random mating, and constant population size.

For a single allelic class, the expected length and height of the coalescence tree up until all lineages in that allelelic class coalesce into a single lineage is equal to the expected length and height of the coalescence tree of the number of individuals in that allelic class. For details, see Hein *et al.* (2005).

$$E[L_{n>1}] = 2N*2\sum_{i=1}^{n-1}\frac{1}{i}$$

$$E[H_{n>1}] = 2N*2(1-\frac{1}{n})$$

To get the total number of expected informative sites in a 1kb region:

$$E[K] = 1000*\mu(T+(T-U))$$

$$E[S] = 1000*\mu(2(U-E[H_{n>1}])+2E[L_{n>1}])$$

The first term in the E[K] expression represents the substitutions due to mutation on the outgroup lineage (chimpanzee in our case) and the last terms, $(T-u)$, comes from substitution occurring along the length of the tree in the ingroup species after speciation but before the start of selection. The first term in E[S] represents the mutations occurring between the start of selection and the coalescence within each allelic class, and the last term comes from polymorphisms originating in the length of the tree during which the lineages within each allelic class have not yet coalesced.

For simplicity, we assume an equilibrium frequency of .5, corresponding to an N_e=5,000 within each allelic class. However, we note that in reality, balancing selection may alter N_e in a complex fashion beyond the scope of this work. We obtain a very similar result with other equilibrium frequencies, due to most of the overall tree length occurring after the last coalescence within each allelic class. We assume a mutation rate of 2.5e-8 mutation events per individual per generation.

$$E[S+K|U=250{,}000] = 22.24$$

$$E[S+K|U=100{,}000] = 18.45$$

Therefore, a total of 20 informative sites, or 10 on each side of the core site, roughly corresponds to looking at a 1kb region in the balanced case. Furthermore, this window size corresponds to an optimal window size of T1 and T2 in a wide variety of conditions **(Fig. S6)**.

· doi:10.1093/molbev/sys000 MBE

DERIVATION OF UNFOLDED θ_B

Let n be the number of chromosomes sampled, d_i be the similarity measure (see main text) and S_i be the number of variants at frequency i in the sample. For ease of calculation, assume no covariance between sites:

$$E[\sum_{i=1}^{n-1} id_iS_i] = \sum_{i=1}^{n-1} E[id_iS_i]$$
$$= \sum_{i=1}^{n-1} id_iE[S_i]$$
$$= \sum_{i=1}^{n-1} id_i\frac{1}{i}\theta$$
$$\hat{\theta}_\beta = \frac{\sum_{i=1}^{n-1} id_iS_i}{\sum_{i=1}^{n-1} d_i}$$

DERIVATION OF FOLDED θ_B

$$E[\sum_{i=1}^{n-1} d_iS_i] = \sum_{i=1}^{n-1} E[d_iS_i]$$
$$= \sum_{i=1}^{n-1} d_iE[S_i]$$
$$= \sum_{i=1}^{n-1} d_i\frac{1}{i}\theta$$
$$\hat{\theta} = \frac{\sum_{i=1}^{n-1} d_iS_i}{\sum_{i=1}^{n-1} d_i\frac{1}{i}}$$

Let $g(x)$ be the folded frequency of a SNP of frequency x, $S_{g(x)}$ be the number of SNPs at that folded frequency, $h = .5(n-1)$ and $m = .5n$. Folding the site frequency spectrum, we obtain:

$$\hat{\theta}_\beta^{fold} = \frac{\sum_{i=1}^{h} d_iS_{g(i)}}{\sum_{i=1}^{h} d_i(\frac{1}{i}+\frac{1}{n-i})} \qquad \text{if n is odd}$$

$$\hat{\theta}_\beta^{fold} = \frac{\sum_{i=1}^{m} d_iS_{g(i)}}{\sum_{i=1}^{m-1} d_i(\frac{1}{i}+\frac{1}{n-i})+d_m\frac{1}{m}} \qquad \text{if n is even}$$

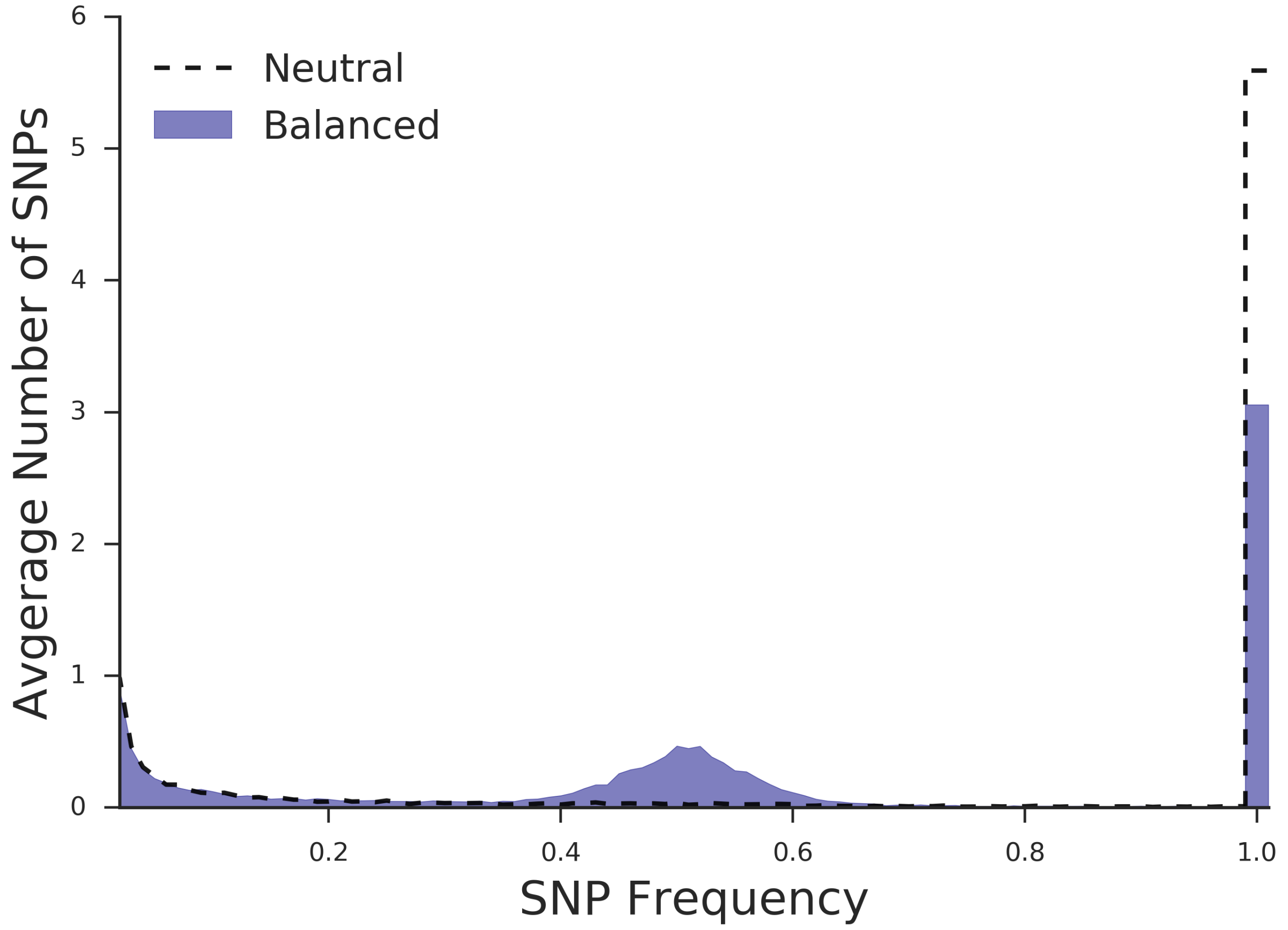

FIG. 1. Site frequency spectrum of derived alleles in balanced or neutral cases, with core variant removed. Window size is 500 base pairs on either side of the core site, with sample size 100 chromosomes. Based on simulations using default parameters and an equilibrium frequency of 0.5.

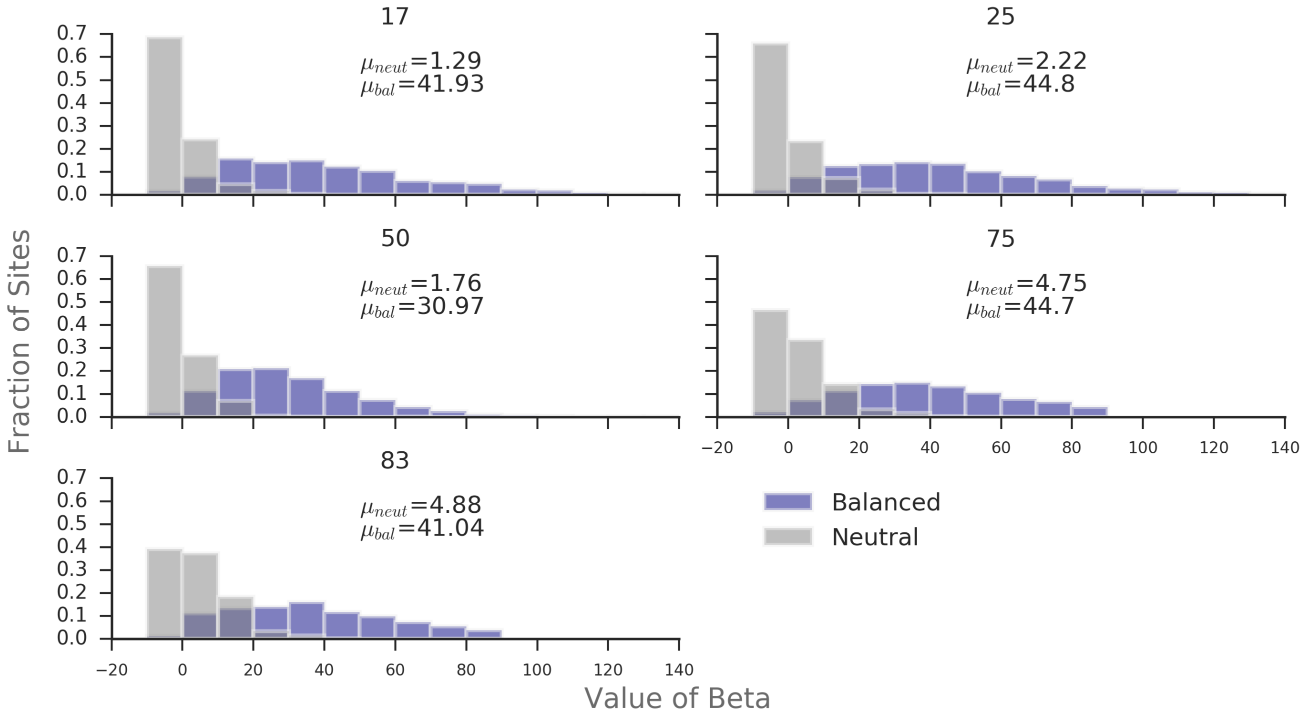

FIG. 2. Distribution of Beta in 1kb windows around a core SNP at different equilibrium frequencies. Based on simulations using default parameters.

· doi:10.1093/molbev/sys000 MBE

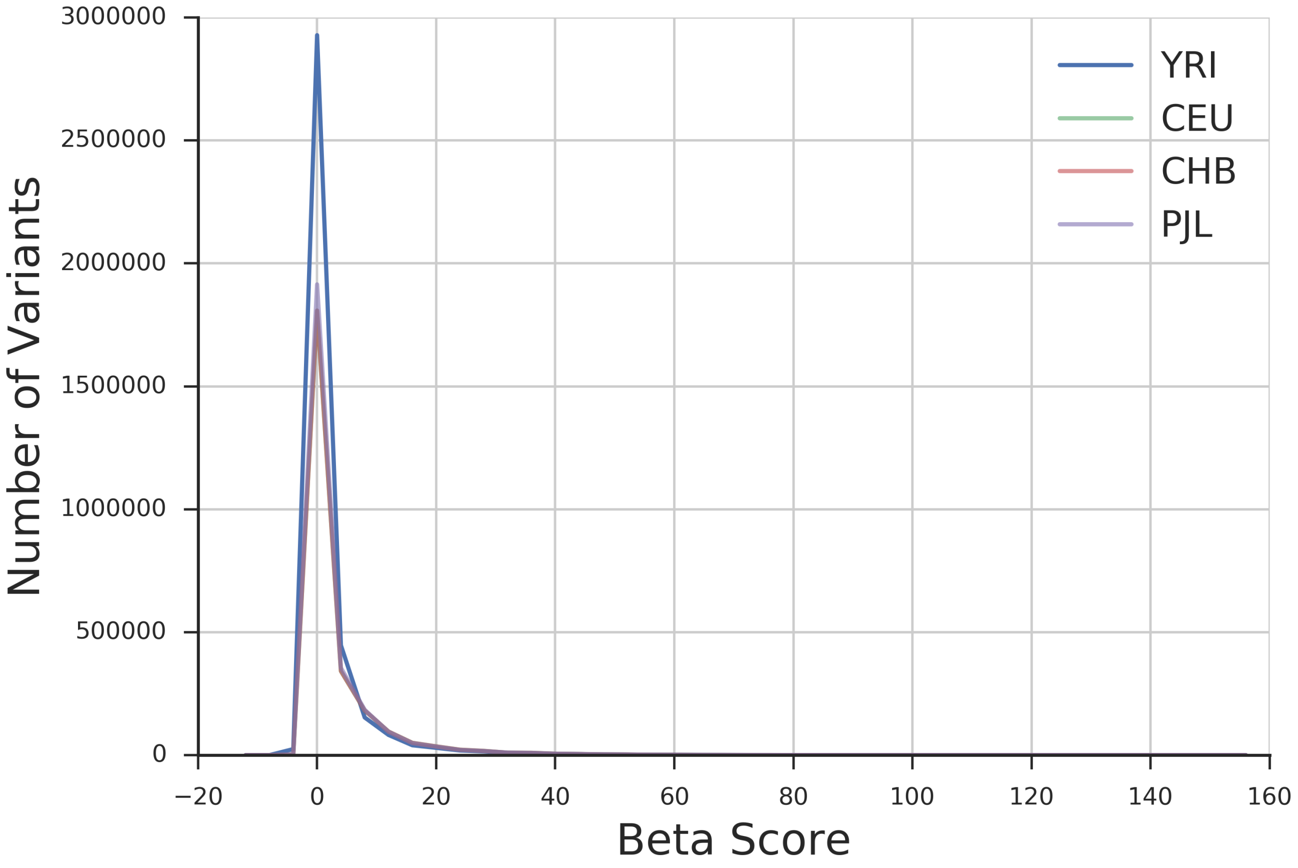

FIG. 3. Distribution of Beta in 4 representative populations. Beta scores binned in units of 4.

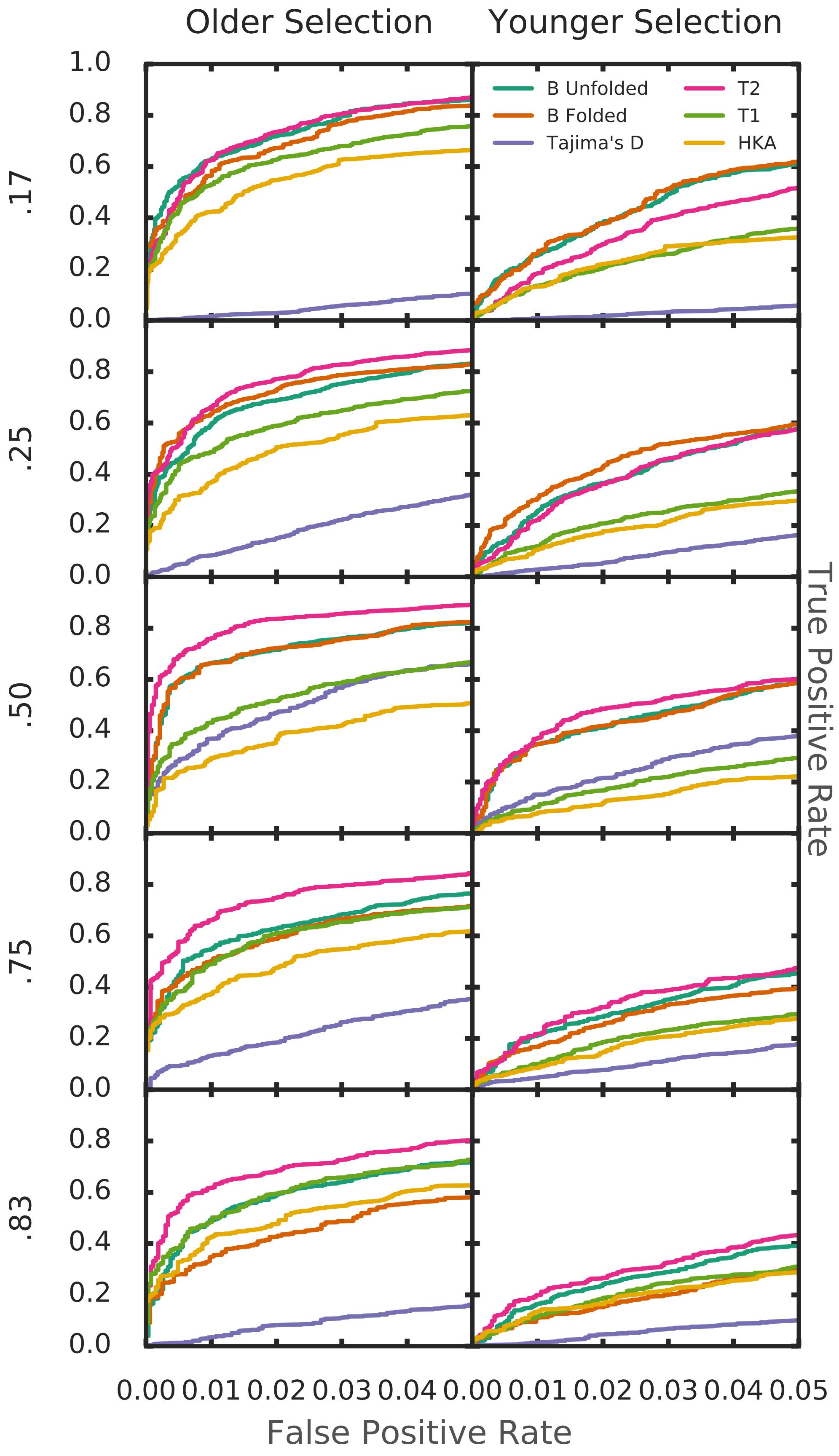

FIG. 4. Power to detect ancient balancing selection under equilibrium demography. Rows correspond to different equilibrium frequencies.

· doi:10.1093/molbev/sys000 MBE

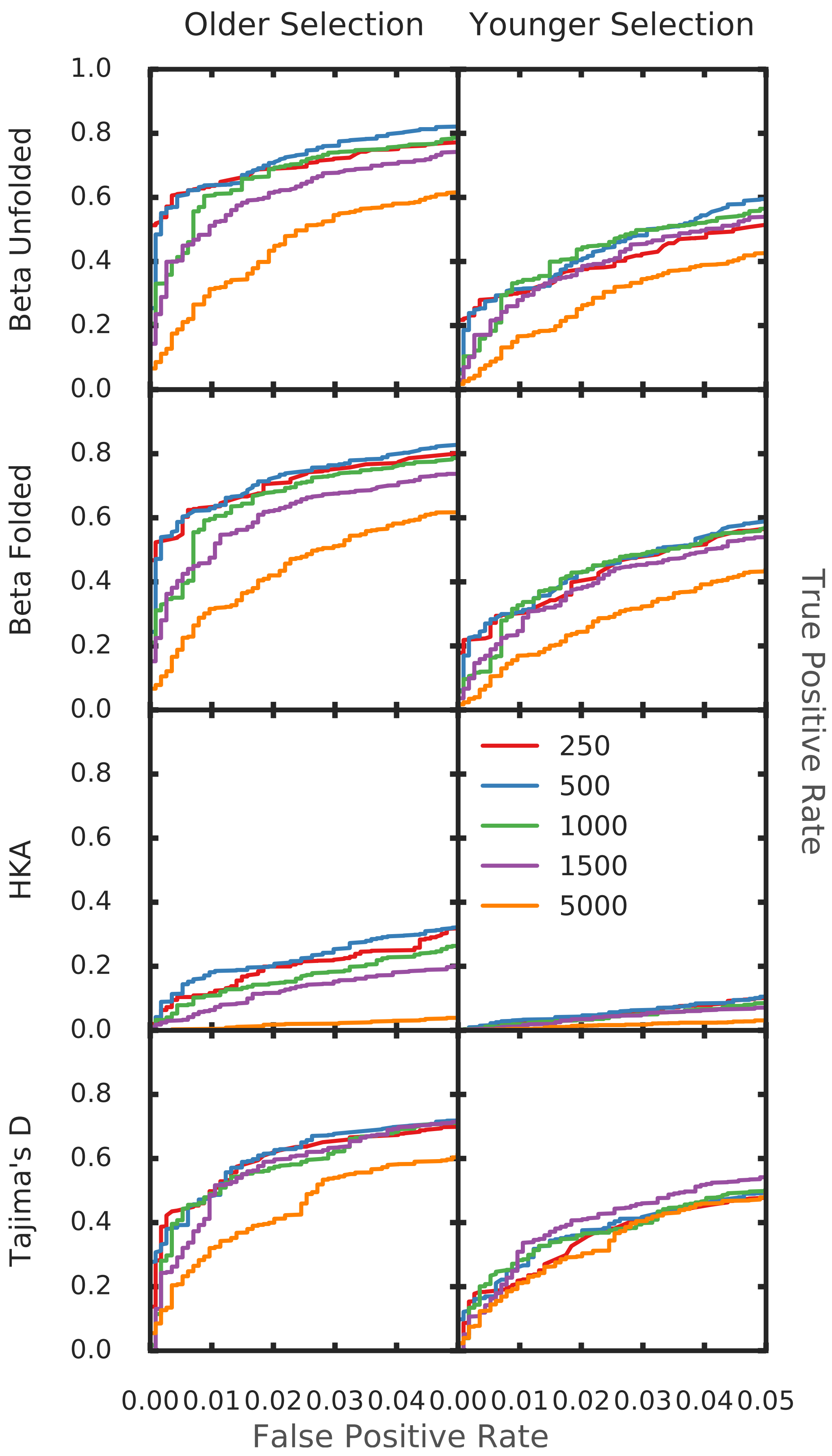

FIG. 5. Power to detect ancient balancing selection using different window sizes, in units of base pairs, with an equilibrium frequency of 0.5.

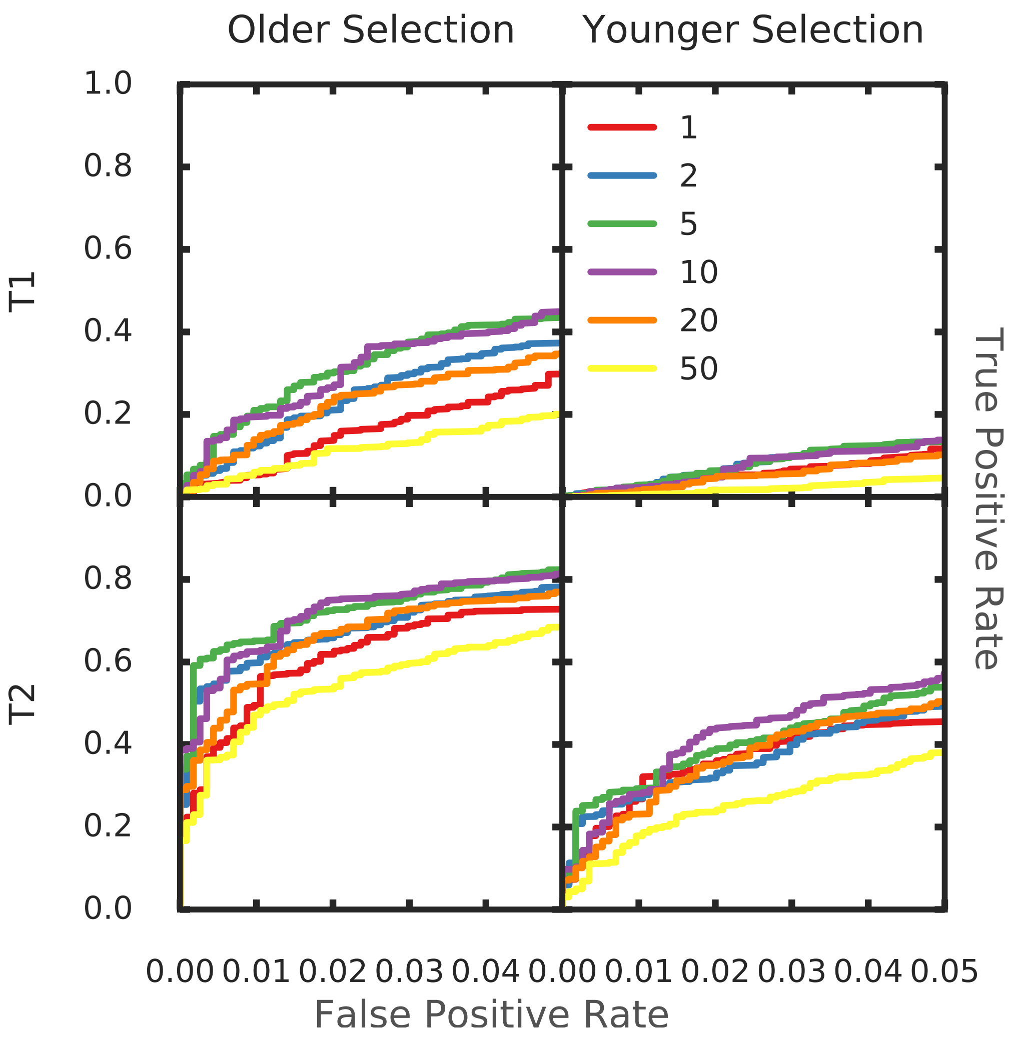

FIG. 6. Power to detect ancient balancing selection using different numbers of informative sites. The number of sites corresponds to the number of sites on either side of the core site.

· doi:10.1093/molbev/sys000 MBE

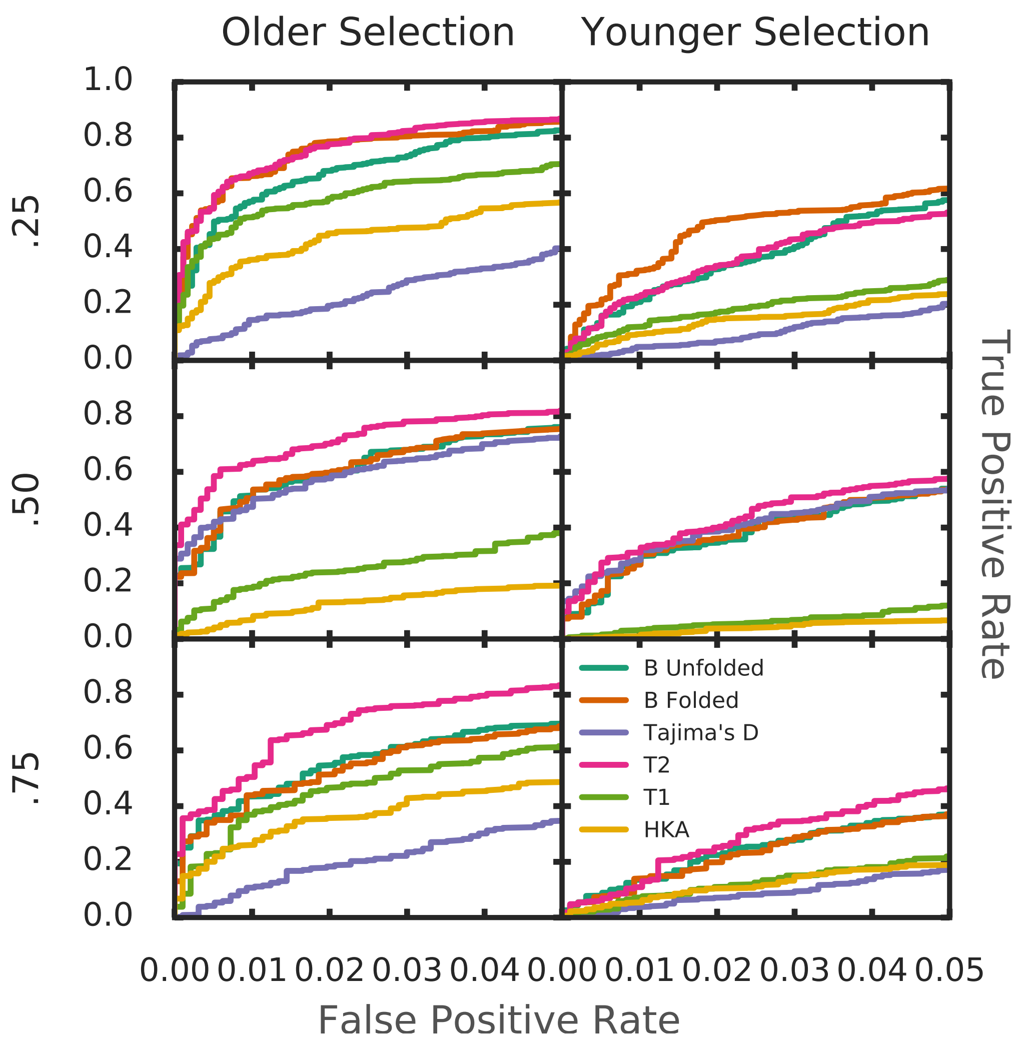

FIG. 7. Power of methods to detect ancient balancing selection under a model of population expansion. In this demographic model, the human population expands to $N_e = 20{,}000$ at generation 302,000, then remains that size until sampling. Based on rescaled simulations.

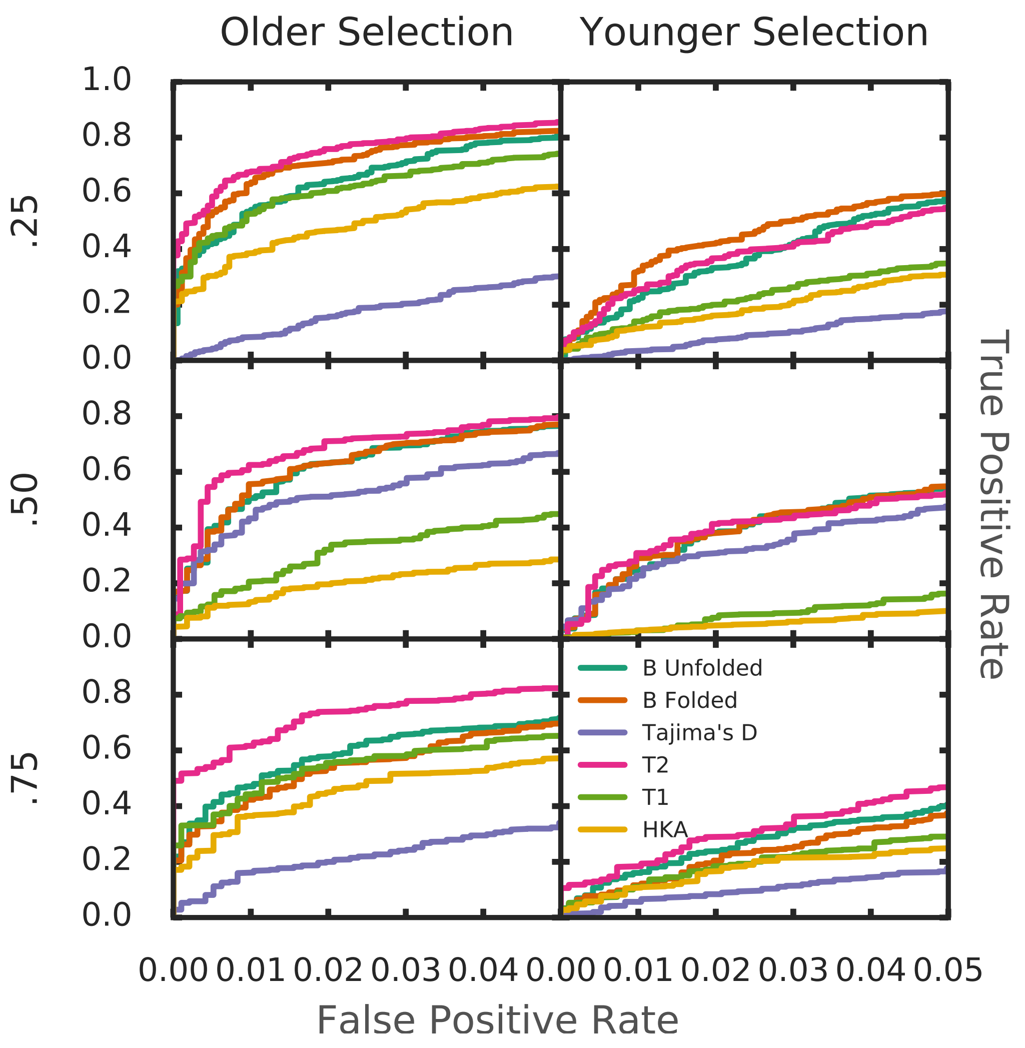

FIG. 8. Power of methods to detect ancient balancing selection under a model of a population bottleneck. Based on rescaled simulations. In this scenario, human population size drops to $N_e = 5{,}500$ from generations 320,000 to 328,000, then returns to $N_e = 10{,}000$. Based on rescaled simulations.

· doi:10.1093/molbev/sys000 MBE

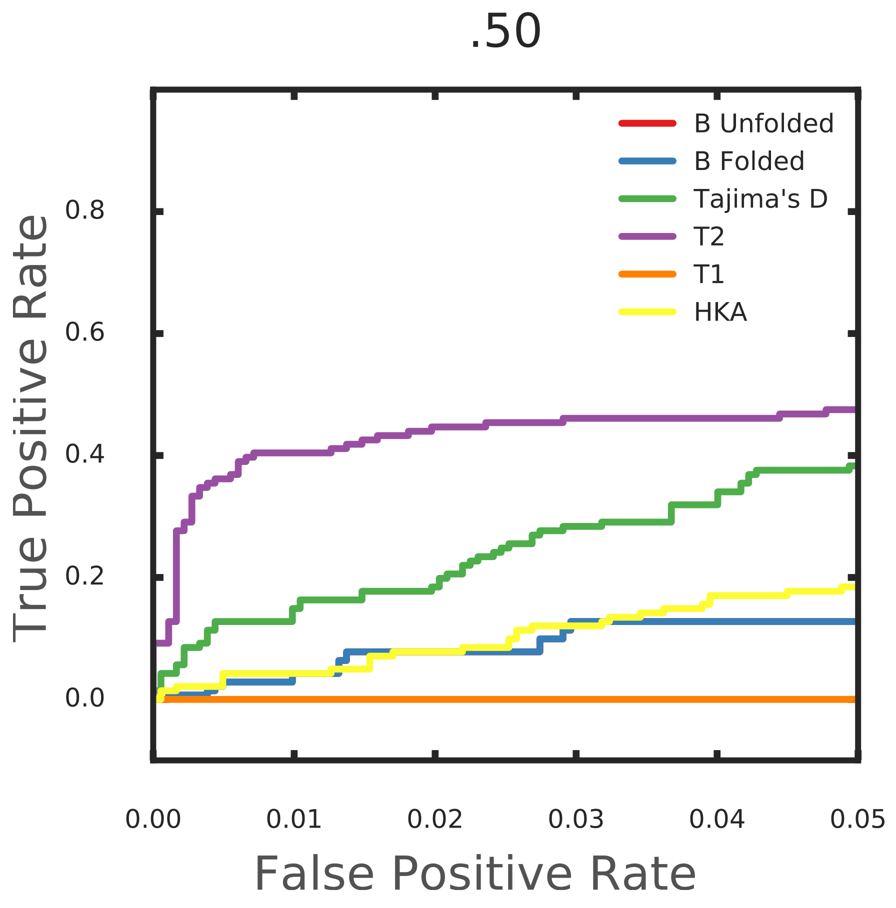

FIG. 9. Power of methods to detect ancient balancing selection under a model of complete population subdivision. In this case, the human population is completely divided into two subpopulations of equal size, $N_e = 5000$, at generation number 300,000, with no admixture between them. The subpopulations were then combined to calculate allele frequencies. This represents an extreme case: there are expected to be a large number of variants at frequency 0.5. In this analysis, we excluded simulation replicates in which the core SNPs was not of frequency exactly .5, in order to investigate the power at the exact frequency that variants are expected to accrue due to population substructure. Balanced variants were of the "older selection" category, so were introduced at generation 100,000. For this analysis, we used the empirical background files from the corresponding neutral simulations, but the estimated divergence time from the simulations using our default rates. This is because the simple divergence time estimator included in BALLET is not able to accurately infer divergence times with the outgroup in the presence of significant population structure. We note that Beta Folded and Unfolded perform nearly identical in this case. Based on rescaled simulations.

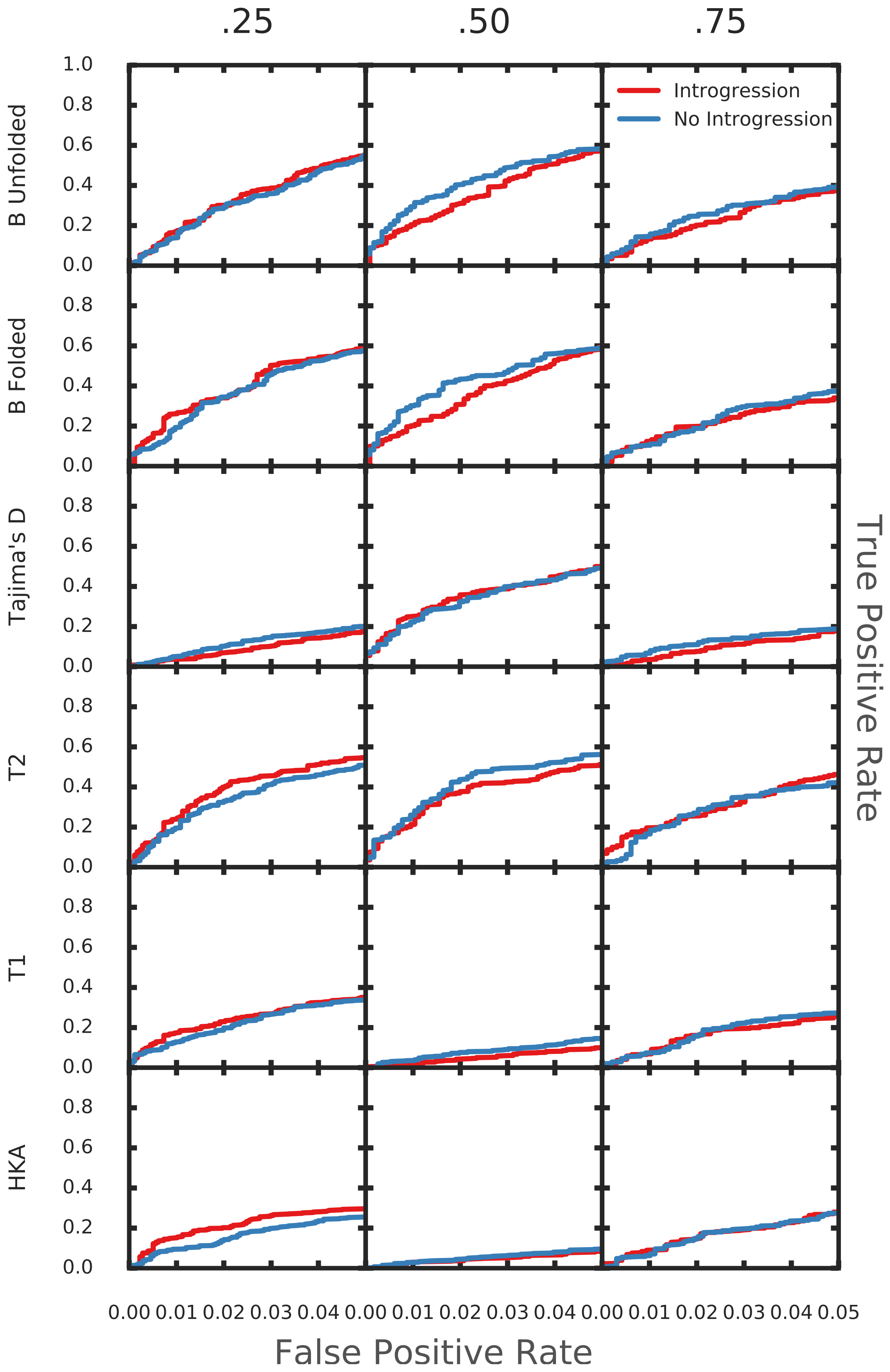

FIG. 10. Power of methods to detect ancient balancing selection under a model of Neanderthal admixture. Balanced variants were of the "younger selection" category, so were introduced at generation 250,000. In this scenario, we simulated Neanderthal admixture into Asian populations. Based on the demographic model presented in Vernot and Akey (2015), we used a two pulse model. We simulated a split from the human lineage into Neanderthal at generation 315,000 with an N_e of 1500. The first pulse of Neanderthal admixture into human occurred from generation 347,750 to 347,780, and had a migration rate of .00075. The second, weaker pulse occurred from generation 347,820 to 347,850, with a migration of .0002. The human N_e remained 10,000 throughout the entire simulation. A chimpanzee population was simulated as before. Based on rescaled simulations.

· doi:10.1093/molbev/sys000 MBE

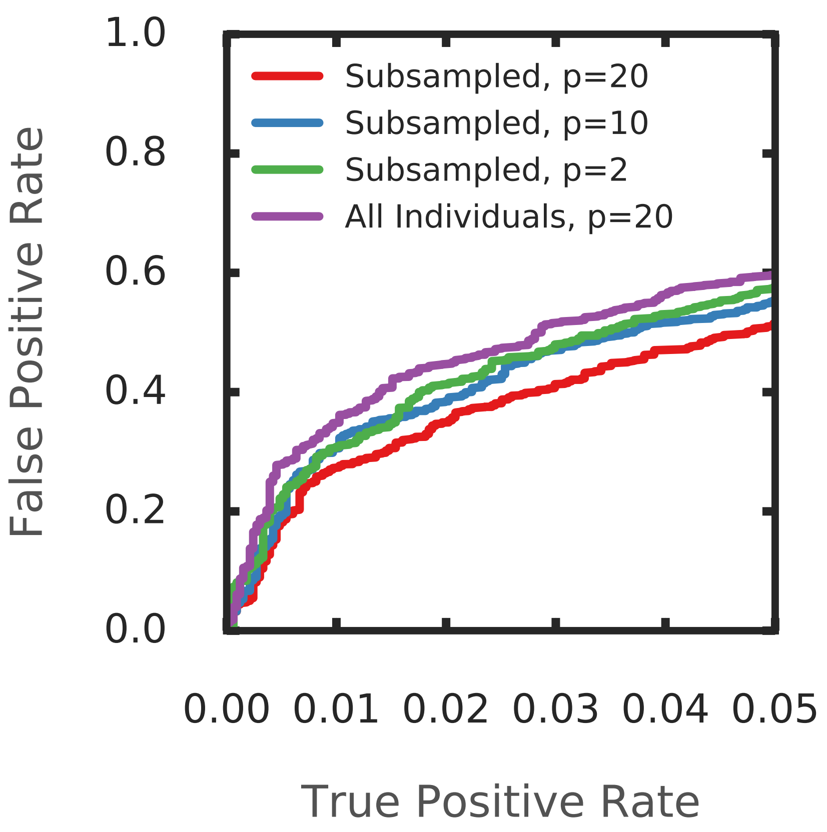

FIG. 11. Power of Beta to detect balancing selection when variant frequencies are calculated using different numbers of individuals. In order to investigate the effects this has on power, we subsampled individuals from our initial set of 100. For each SNP in each simulation replicate, we chose a number uniformly, between 0 and 25, of individuals to remove. After these individuals were removed the frequency was recalculated on the remaining individuals.

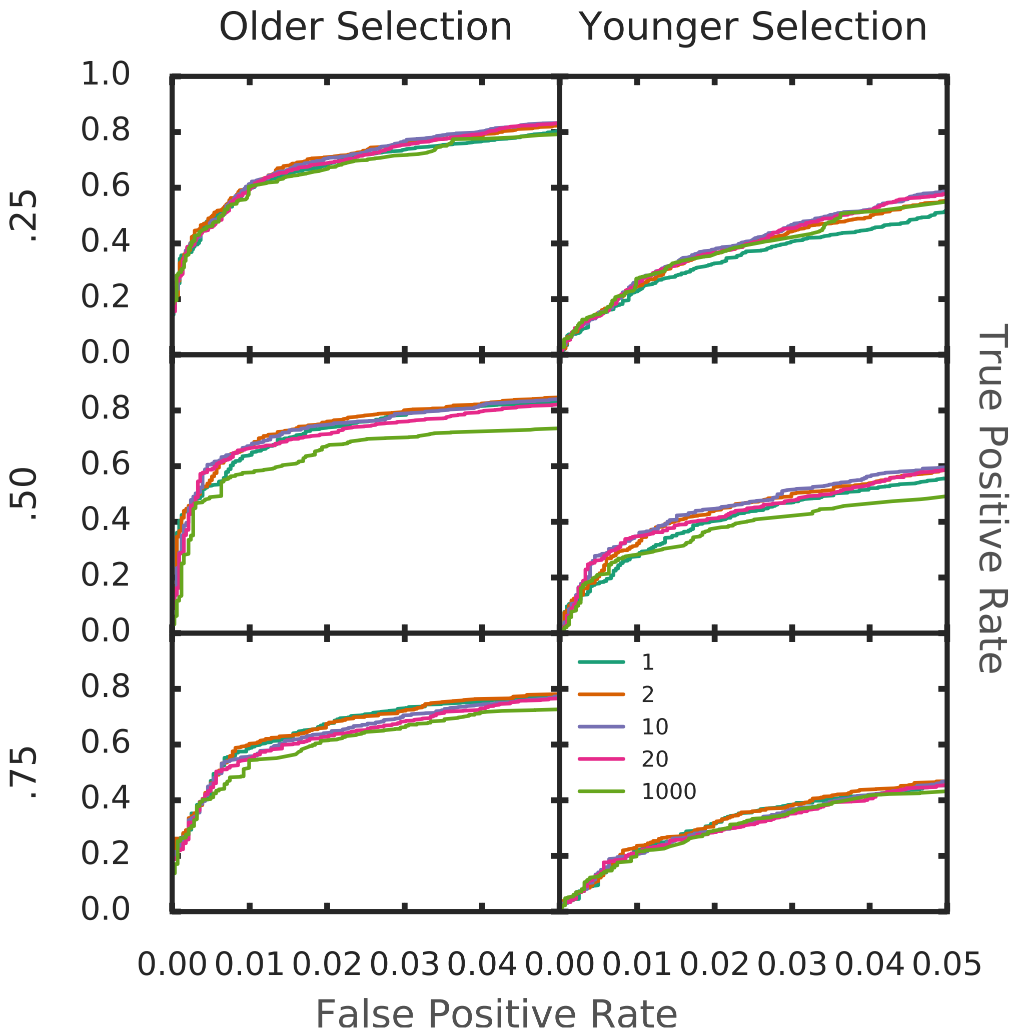

FIG. 12. Power of methods to detect ancient balancing selection using different value of p parameter with Beta

· doi:10.1093/molbev/sys000 MBE

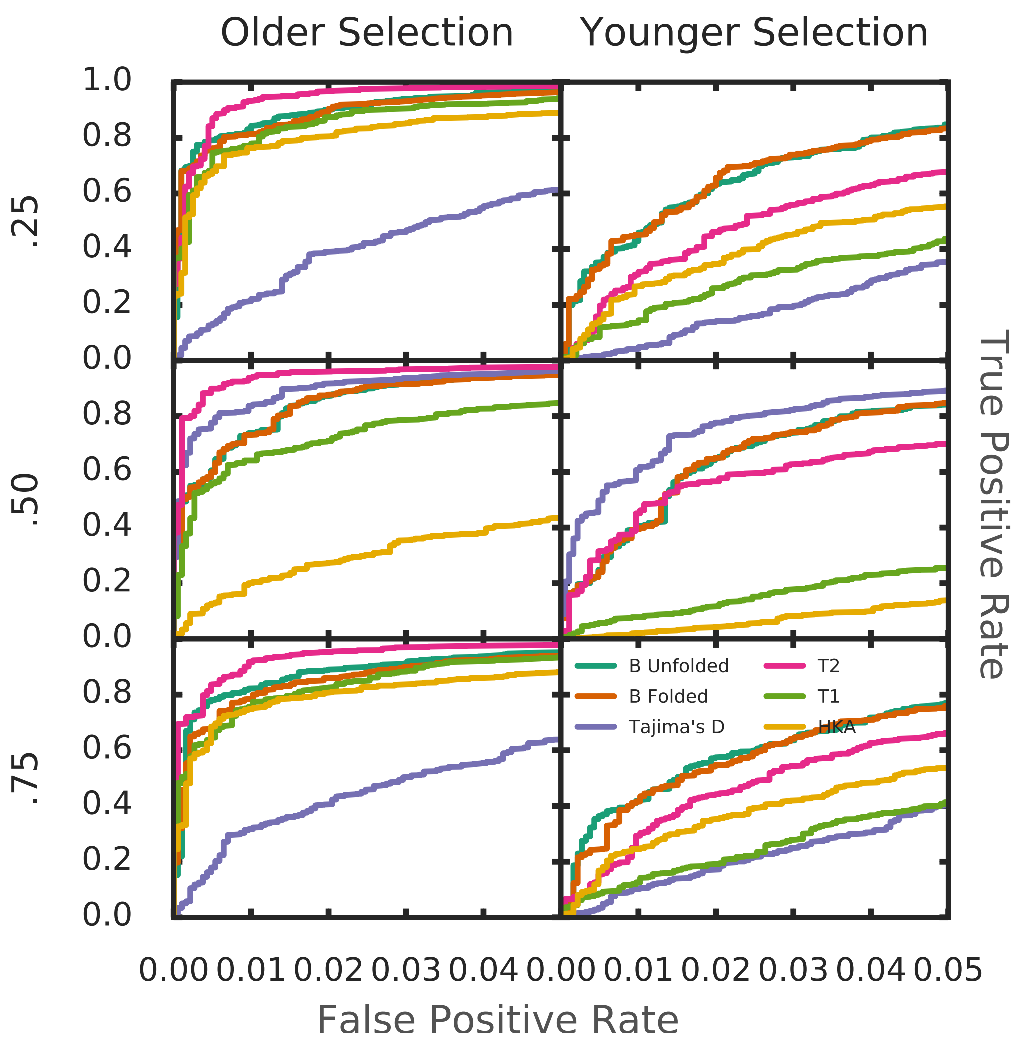

FIG. 13. Power of methods with an increased mutation rate of 2.5e-7. Based on rescaled simulations.

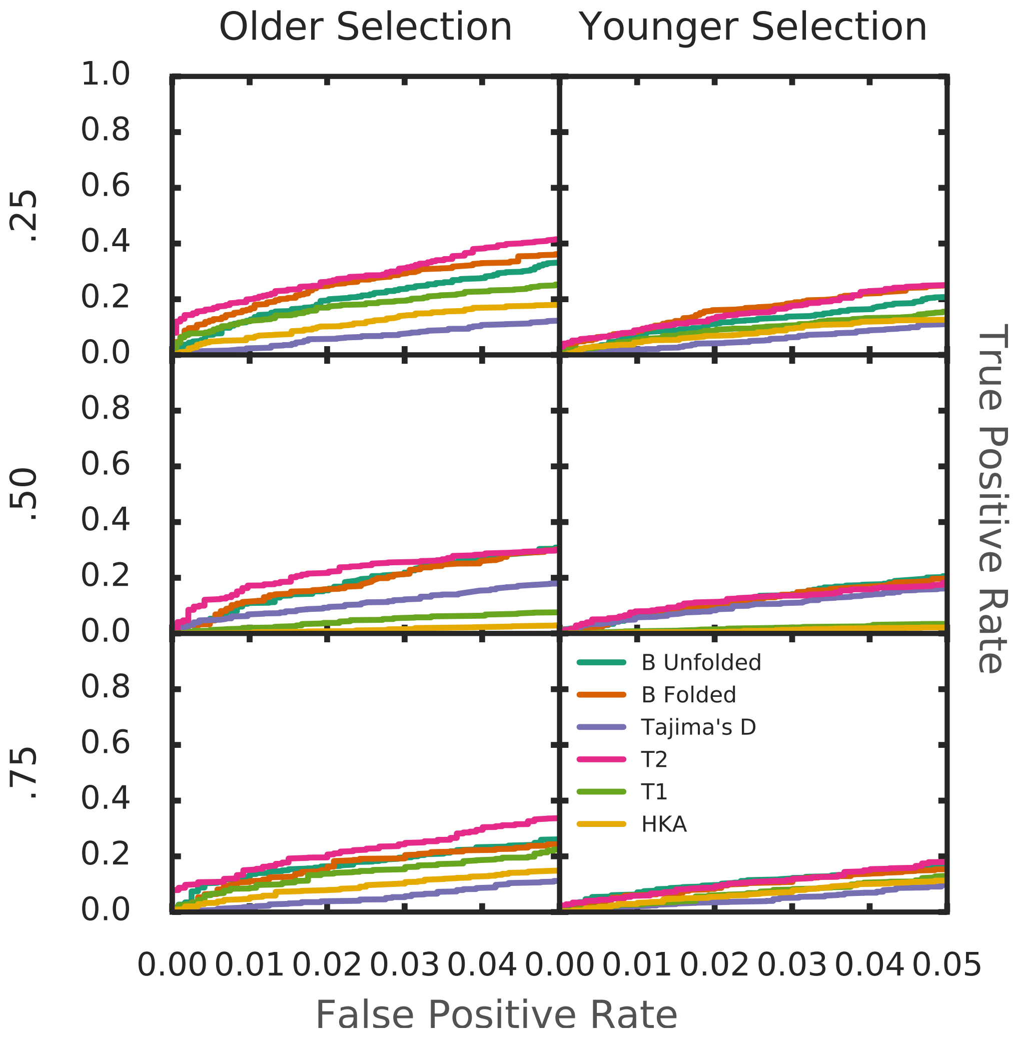

FIG. 14. Power of methods with an increased recombination rate of 2.5e-7. Based on rescaled simulations.

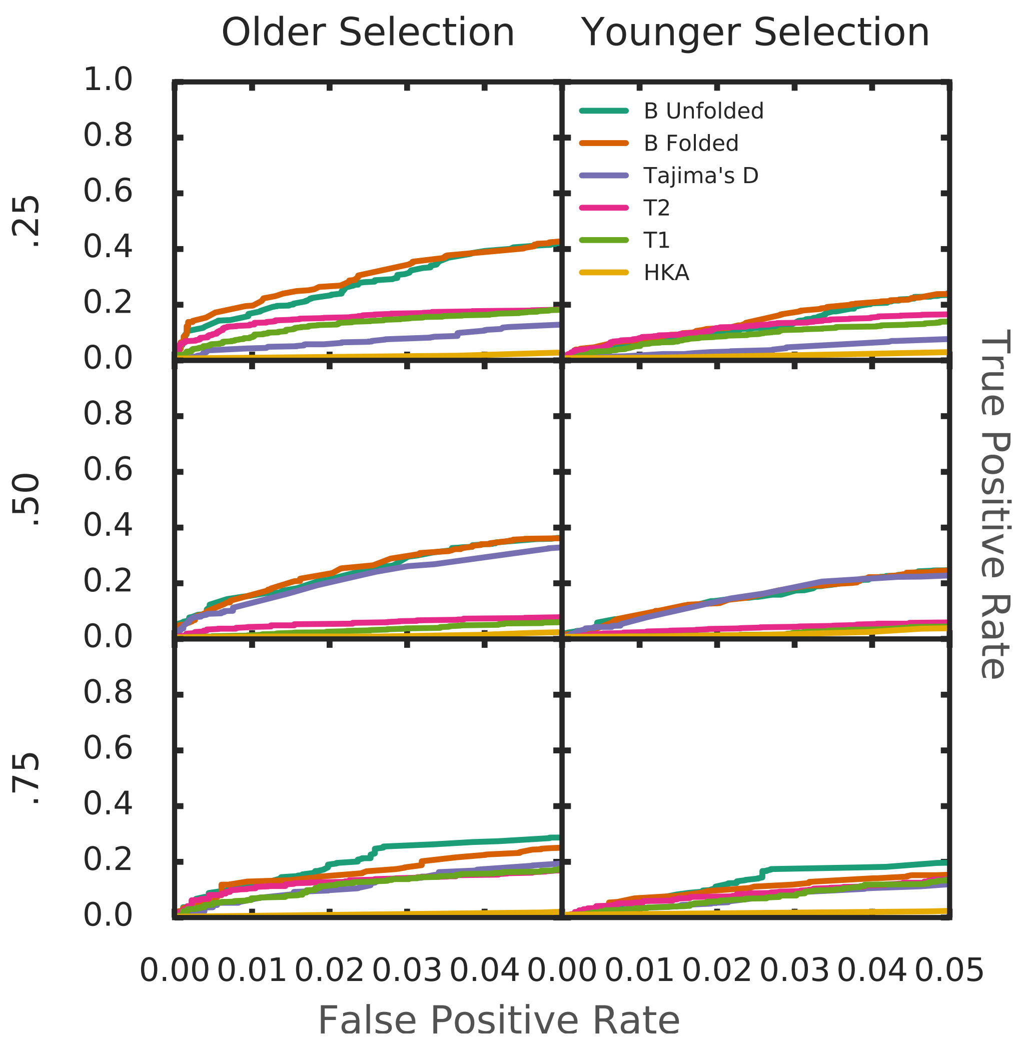

FIG. 15. Power of methods with a decreased mutation rate of 2.5e-9. We note that T1 and T2 perform poorly due to there not being 20 informative sites in the 10 kb simulated region, which results in an error. Based on rescaled simulations.

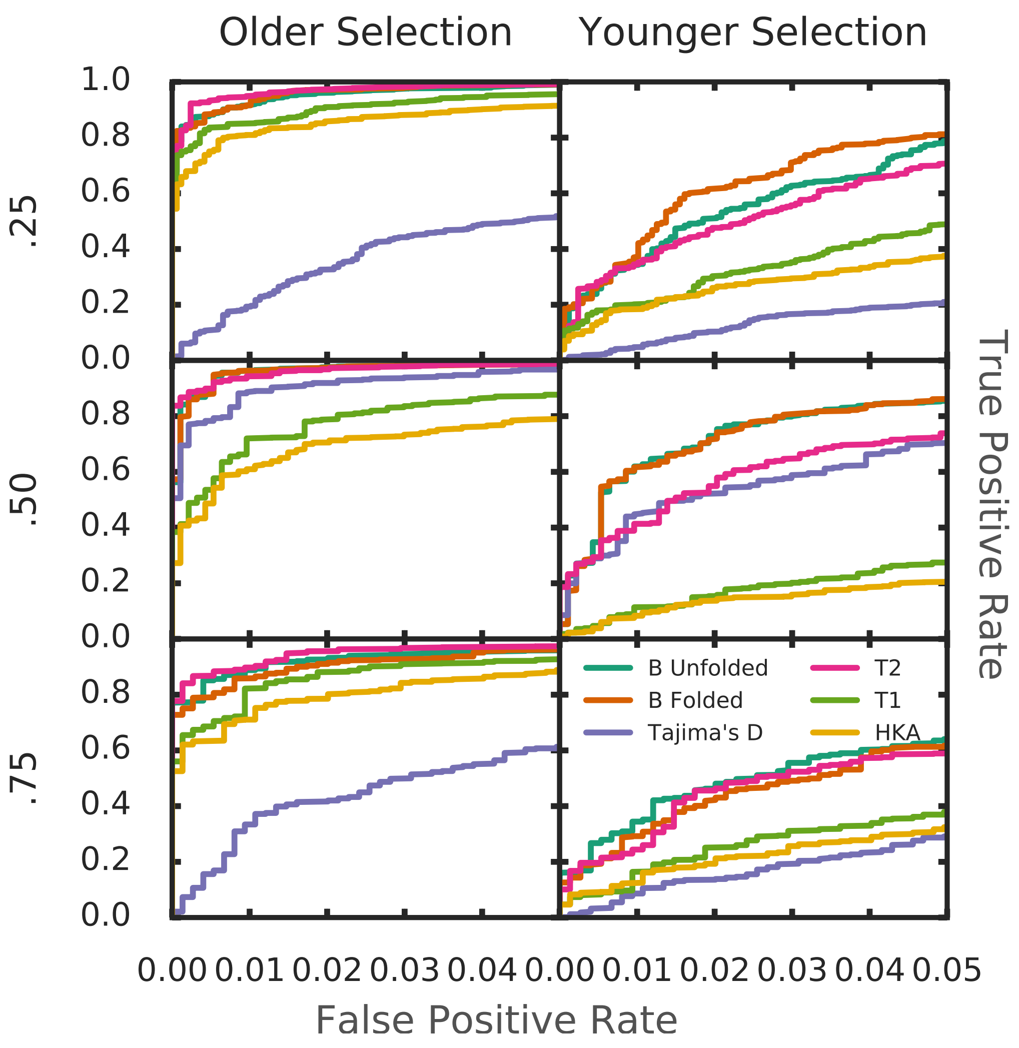

FIG. 16. Power of methods with a decreased recombination rate of 2.5e-9

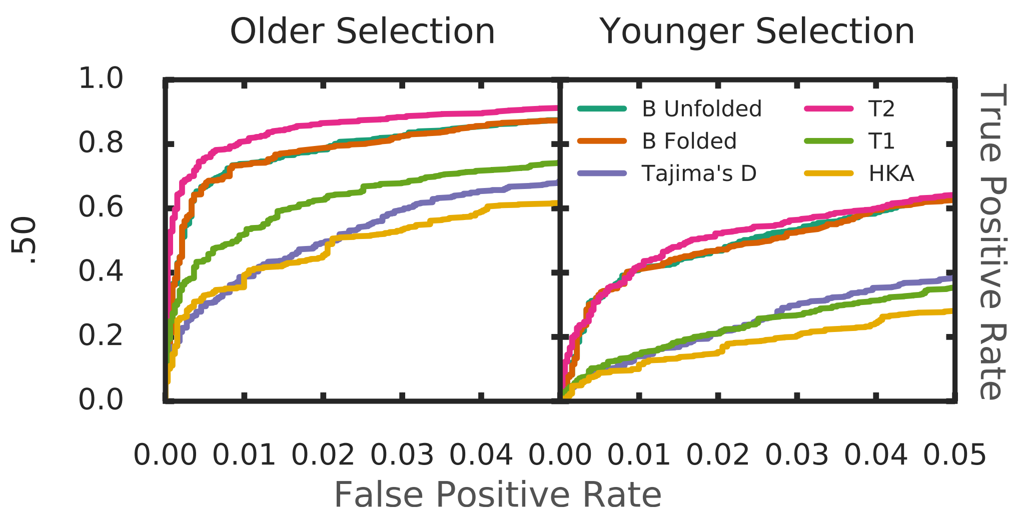

FIG. 17. Power of methods with a selective coefficient of 1e-4 and overdominance coefficient of h=100. We were only able to test power with $h=100$, because of the extremely high frequency at which the balanced allele was lost at other equilibrium frequencies.

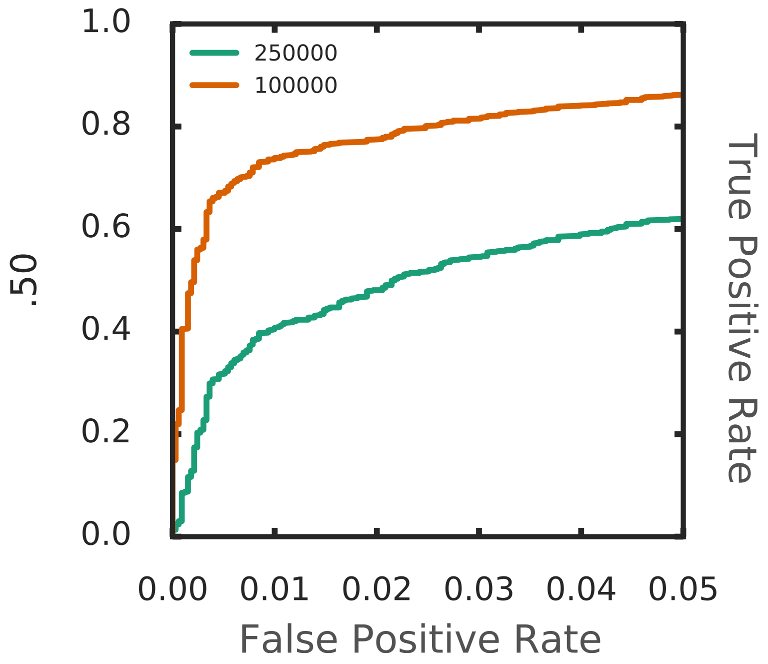

FIG. 18. Power of the unfolded Beta statistic under a model of frequency-dependent selection. In this case, the fitness coefficient was .01 and the overdominance coefficient was .05. The fitness of the derived allele was set to equal 1.5 minus the frequency of the allele. This results in an equilibrium frequency of .5. The color corresponds to age of selection, either 100,000 generations after the start of selection (older selection) or 250,000 generations after the start(younger selection).

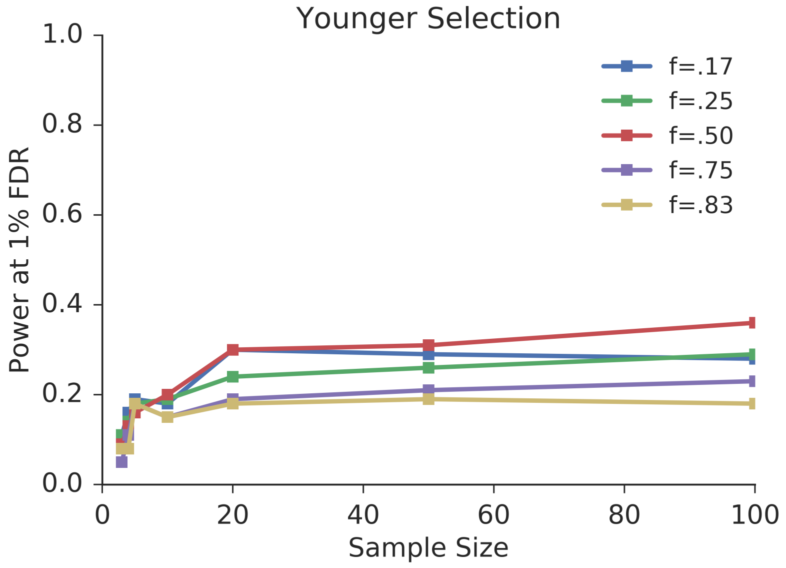

FIG. 19. Power of β at a 1 percent false discovery rate to detect selection 100,000 generations old, by number of chromosomes sampled.

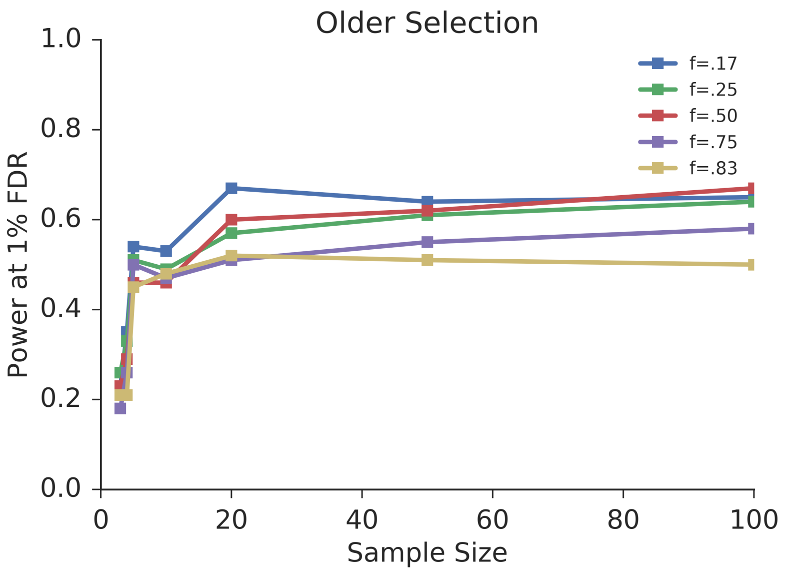

FIG. 20. Power of β at a 1 percent false discovery rate to detect selection 250,000 generations old, by number of chromosomes sampled.

· doi:10.1093/molbev/sys000 MBE

References

DeGiorgio, M., Lohmueller, K. E., and Nielsen, R. 2014. A Model-Based Approach for Identifying Signatures of Ancient Balancing Selection in Genetic Data. *PLoS Genetics*, 10(8): e1004561.

Gao, Z., Przeworski, M., and Sella, G. 2015. Footprints of ancient-balanced polymorphisms in genetic variation data from closely related species. *Evolution*, 69(2): 431–446.

Hein, J., Schierup, M. H., and Wiuf, C. 2005. *Gene genealogies, variation and evolution : a primer in coalescent theory*. Oxford University Press.

Vernot, B. and Akey, J. M. 2015. Complex history of admixture between modern humans and Neandertals. *American journal of human genetics*, 96(3): 448–53.
